# Childhood-onset systemic lupus erythematosus presenting with growth retardation and delayed puberty: a case report

**DOI:** 10.3389/fped.2026.1822737

**Published:** 2026-05-25

**Authors:** Yumo Liu, Xinyu Li, Zhengnan Gao, Xuhan Liu

**Affiliations:** 1 Department of Endocrinology, Dalian Municipal Central Hospital Affiliated to Dalian University of Technology, Dalian, China; 2 Dalian Medical University, Dalian, China

**Keywords:** childhood-onset systemic lupus erythematosus, connective tissue disorders, delayed puberty, growth retardation, malnutrition

## Abstract

Childhood-onset systemic lupus erythematosus (cSLE) is a chronic, multisystem autoimmune disease characterized by marked clinical heterogeneity and substantial morbidity. Although rash, arthritis, and renal involvement are common manifestations, atypical presentations may delay diagnosis. In rare cases, growth retardation and delayed puberty may precede typical systemic features, posing a diagnostic challenge. We report the case of a 12-year-old Chinese girl who presented to the endocrinology clinic with progressive growth retardation and delayed pubertal development. Comprehensive growth assessment demonstrated discordance among height velocity, weight gain, and pubertal progression. Further evaluation revealed proteinuria, hypoalbuminemia, generalized lymphadenopathy, and immunological abnormalities, leading to a diagnosis of cSLE. Following immunosuppressive therapy, systemic manifestations improved and laboratory indices gradually normalized; however, longitudinal follow-up showed that impairment in growth and pubertal progression persisted despite disease control. This case highlights that cSLE, although a rare cause of growth retardation and delayed puberty, should be considered in adolescents with unexplained short stature or pubertal delay, particularly when accompanied by proteinuria or immunological abnormalities. Early recognition and multidisciplinary management are essential to improve long-term growth and overall clinical outcomes.

## Introduction

Systemic lupus erythematosus (SLE) is a chronic, multisystem autoimmune disease with heterogeneous clinical manifestations (1, 2). Childhood-onset SLE (cSLE), defined as disease onset before 18 years of age, accounts for approximately 15%–20% of all SLE cases and represents the second most common systemic connective tissue disease in children. In Western populations, the annual incidence ranges from 0.36 to 2.5 per 100,000, with a prevalence of 1.89–25.7 per 100,000 (3); precise epidemiological data remain limited in China. Compared with adult-onset SLE, cSLE is typically associated with more acute onset, higher disease activity, and more severe organ involvement, particularly renal and neuropsychiatric manifestations (4, 5). The peak age at onset is 10–13 years, with approximately 85% of cases occurring after 8 years of age, and a marked female predominance (approximately 6:1) (6).

Most pediatric patients present with characteristic features such as malar rash, arthritis, nephritis, or systemic symptoms; however, in rare cases, cSLE may initially manifest as growth retardation or delayed puberty (7). Such atypical presentations are easily misdiagnosed as constitutional delay of growth and puberty or as primary endocrine disorders. Growth impairment in cSLE is multifactorial and may be related to chronic inflammation, glucocorticoid exposure, and disruption of the GH–IGF-1 and hypothalamic–pituitary–gonadal axes (8, 9), often accompanied by delayed bone age and hypoestrogenism, which may compromise final adult height (10). Here, we report a girl with cSLE who initially presented with delayed puberty and short stature, highlighting that cSLE, although rare, should be considered in the differential diagnosis of unexplained growth retardation or pubertal delay, particularly when accompanied by proteinuria or immunological abnormalities.

## Case report

**Figure 1:**
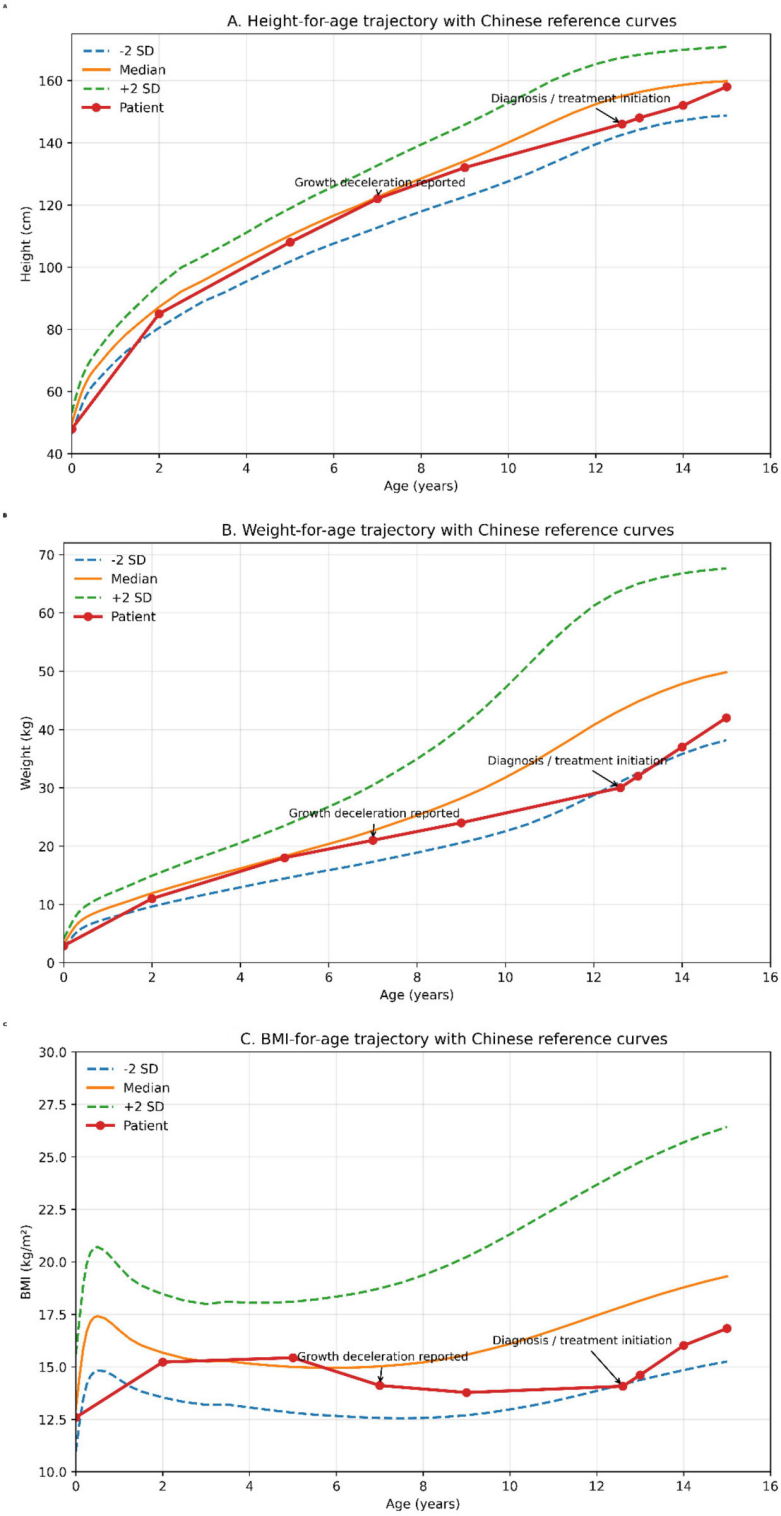
Longitudinal growth trajectories of the patient plotted against Chinese reference curves. **(A)** Height-for-age. **(B)** Weight-for-age. **(C)** BMI-for-age. Serial measurements before disease manifestation, at presentation, and during follow-up were plotted against Chinese reference curves for girls (−2 SD, median, and +2 SD). Growth deceleration was noted from 7 years of age. The patient was diagnosed with childhood-onset systemic lupus erythematosus at 12.6 years of age, after which height velocity, weight gain, and BMI gradually improved during follow-up.

A 12-year-7-month-old girl was referred to the endocrinology clinic for progressive growth deceleration since the age of 7 years and absence of pubertal development. She had no history of chronic illness, long-term medication use, recurrent oral or genital ulcers, or musculoskeletal complaints. There were no abnormalities in the patient's birth history or early developmental milestones. According to the parents’ account and the available school health records, linear growth was reportedly normal until age 6; thereafter, the rate of height gain gradually slowed, and weight gain leveled off. No secondary sexual characteristics had appeared within the year prior to the current visit. Longitudinal growth trajectories of the patient plotted against Chinese reference curves are presented in Figure 1 (11). Her father is 175 cm tall and weighs 80 kg (BMI 26.1 kg/m^2^), while her mother is 162 cm tall and weighs 50 kg (BMI 19.0 kg/m^2^). Based on the CMH (the Corrected Midparental Height) method, the child's target height is 162 cm. The mother reported that her age at menarche was 13 years, and there is no family history of delayed puberty, short stature, autoimmune diseases, or endocrine disorders. Physical examination: her height was 146 cm (−2 to −1 standard deviations), weight 30 kg (<−2 standard deviations), and BMI 14.1 kg/m^2^ (<−2 standard deviations). Tanner staging showed breast stage I and pubic hair stage I. No dysmorphic features, goiter, or skeletal abnormalities were observed. However, multiple firm, non-tender lymph nodes were palpable in the cervical, supraclavicular, and axillary regions.

The initial evaluation focused primarily on endocrine causes of short stature and delayed puberty, including constitutional delay of growth and puberty, hypogonadotropic hypogonadism, hypergonadotropic hypogonadism, and chronic systemic disease. Assessment of growth, nutritional status, and skeletal maturity showed that the patient presented with short stature and delayed pubertal development. Preliminary assessment indicated that, according to national growth standards, height fell between −2 and −1 standard deviations, while weight was below −2 standard deviations. The BMI was 14.1 kg/m^2^, which was below −2 standard deviations, suggesting undernutrition. Body proportions were normal, with no signs of deformity. Bone age was delayed by approximately 1.5 years compared with chronological age. Longitudinal growth velocity required further dynamic assessment. Breast ultrasonography demonstrated bilateral subareolar hypoechoic glandular tissue, consistent with early thelarche. Pelvic ultrasonography showed a uterine length >4 cm with visible endometrial echo and ovarian volumes >3 mL containing multiple follicles ≥4 mm in diameter. These findings support estrogen exposure and pubertal onset. However, ultrasonographic findings are adjunctive rather than diagnostic, and biochemical confirmation by stimulation testing remains the key evidence for activation of the hypothalamic–pituitary–gonadal axis. A triptorelin (LHRH*α*) stimulation test (12) was performed after an overnight fast. Following intramuscular administration of 100 μg triptorelin, blood samples were collected at 0, 15, 30, 45, 60, and 90 min to measure LH and FSH levels using [electrochemiluminescence]. In our laboratory, a peak LH level >18 mIU/mL is considered consistent with pubertal activation in girls. In this patient, the peak LH level was 35.24 mIU/mL at 30 min, indicating a pubertal response pattern and confirming activation of the HPG axis, thereby making hypogonadotropic hypogonadism unlikely. In addition, pelvic ultrasonography demonstrated uterine and ovarian development, which, together with the gonadotropin profile, made Turner syndrome unlikely. Baseline biochemical, hematologic, and endocrine findings are summarized in Table 1. Physical examination showed Tanner stage B1P1. Although external pubertal signs were minimal, both hormonal and imaging findings supported partial activation of the hypothalamic–pituitary–gonadal axis. Taken together, and in accordance with the Chinese Expert Consensus on Clinical Practice for the Assessment and Management of Physical Development in Children (13), these findings suggest growth impairment associated with nutritional deficiency rather than isolated constitutional delay of growth and puberty (14).

**Table 1:** Laboratory investigations.

Parameter	Result	Reference range
Hematology & coagulation		
WBC ( × 10⁹/L)	5.15	4.3–11.3
Neutrophils ( × 10⁹/L)	2.9	1.6–7.8
Neutrophil %	56.4	31–70
Lymphocyte %	32.4	23–59
Monocyte %	8.5	2–11
Eosinophil %	2.4	0–9
Basophil %	0.3	0–1
Hemoglobin (g/L)	123	118–156
Platelets ( × 10⁹/L)	170	150–407
Biochemistry		
ALT (U/L)	21	7–30
AST (U/L)	32	14–44
Total protein (g/L)	61.2	65–84
Albumin (g/L)	30.5	39–54
Total bilirubin (µmol/L)	6.1	≤21
Direct bilirubin (µmol/L)	1.4	<3.4
ALP (U/L)	95	81–454
BUN (mmol/L)	3.57	2.5–6.5
Creatinine (µmol/L)	31	27–66
Uric acid (µmol/L)	326	155–357
LDH (U/L)	193	120–250
K⁺ (mmol/L)	3.88	3.7–5.2
Na⁺ (mmol/L)	139.9	135–145
Ca^2^⁺ (mmol/L)	2.10	2.10–2.80
Inorganic P (mmol/L)	1.63	1.03–1.86
Mg^2^⁺ (mmol/L)	0.78	0.70–1.00
Total cholesterol (mmol/L)	4.50	<5.18
Triglycerides (mmol/L)	3.25	<1.70
HDL (mmol/L)	0.54	1.04–1.55
LDL (mmol/L)	2.44	<3.37
Bone & nutritional markers		
PTH (pg/mL)	48.8	18.5–88
25(OH)D (ng/mL)	16.41	>30
Osteocalcin (ng/mL)	37.7	–
*β*-CTX (ng/mL)	1.296	–
P1NP (ng/mL)	246.7	–
Glucose metabolism		
Fasting glucose (mmol/L)	4.19	3.9–6.1
Insulin (mU/L)	4.0	3–25
C-peptide (ng/mL)	0.45	0.81–3.85
Urine		
Protein	Positive	Negative
Pituitary–thyroid axis		
TSH (mIU/L)	1.103	0.38–4.34
FT3 (pmol/L)	5.19	2.77–6.31
FT4 (pmol/L)	15.42	10.44–24.38
TgAb (U/mL)	338.68	<4.5
TPOAb (U/mL)	59.81	0–60
Pituitary–adrenal axis		
ACTH (pg/mL)	18.25	7–65
Cortisol 08:00 (µg/dL)	10.98	–
Cortisol 16:00 (µg/dL)	5.62	–
Cortisol 00:00 (µg/dL)	11.92	–
Overnight 1-mg DST cortisol 08:00 (µg/dL)	0.72	–
Pituitary–gonadal axis		
LH (mIU/mL)	12.15	–
FSH (mIU/mL)	11.55	–
Estradiol (nmol/L)	80.20	–
Testosterone (nmol/L)	0.15	–
Progesterone (nmol/L)	0.38	–
Other hormones		
GH (ng/mL)	6.30	0–8.05
IGF-1 (ng/mL)	76.50	60–350
Androstenedione (nmol/L)	0.91	<7.71
DHEA-S (µg/dL)	20.25	34–280.55
DHEA (ng/mL)	0.47	1.2–6.3
PRL (ng/mL)	26.70	3.4–24.1
DHT (pg/mL)	<9.4	22.5–280.6
Free testosterone (pg/mL)	0.83	0–4.2
SHBG (nmol/L)	84.05	12.9–134.9
17-OHP (ng/mL)	0.19	<2.32
β-HCG (mIU/mL)	<0.1	0–3

HDL, high-density lipoprotein; LDL, low-density lipoprotein; β-CTX, beta C-terminal telopeptide of type I collagen; P1NP, procollagen type I N-terminal propeptide; β-HCG, beta human chorionic gonadotropin. “–” indicates that a reference range was not available. TSH, thyroid-stimulating hormone; FT3, free triiodothyronine; FT4, free thyroxine; TgAb, thyroglobulin antibody; TPOAb, thyroid peroxidase antibody; ACTH, adrenocorticotropic hormone; LH, luteinizing hormone; FSH, follicle-stimulating hormone; GH, growth hormone; IGF-I, insulin-like growth factor I; PRL, prolactin; DHEA-S, dehydroepiandrosterone sulfate; DHT, dihydrotestosterone; SHBG, sex hormone-binding globulin; 17-OHP, 17-hydroxyprogesterone.

**Figure 2:**
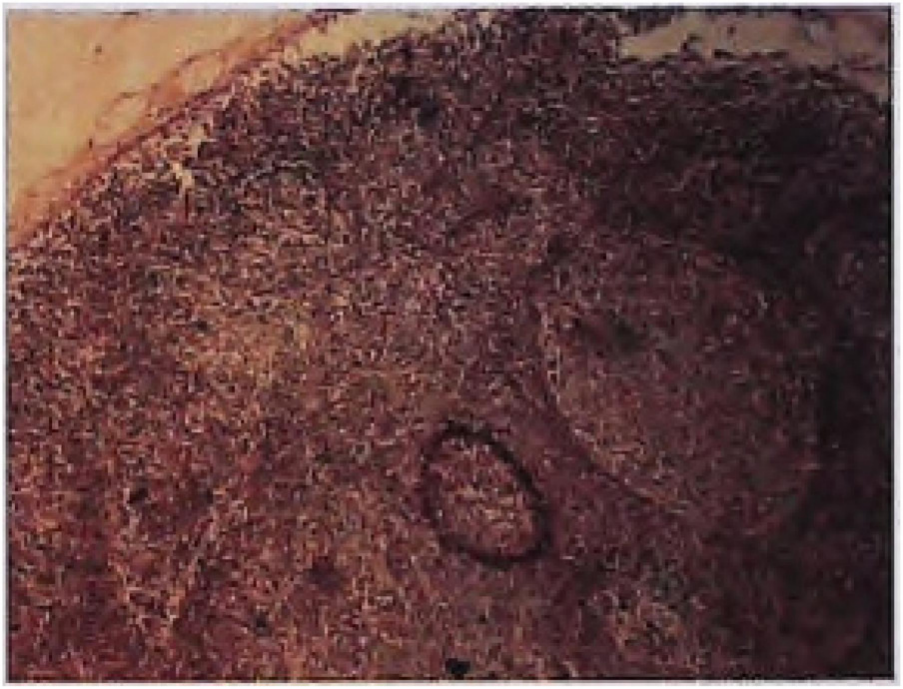
Pathological diagnosis: (neck) lymph node: lymphoid follicular hyperplasia. Immunohistochemistry reveals significant plasma cell infiltration within the germinal centers. Secondary lymph node changes associated with autoimmune disorders cannot be excluded; correlate with clinical presentation. Immunohistochemistry: 2. CD20 (B-cell positive), CD19 (B-cell positive), CD10 (germinal center positive), Bcl-6 (germinal center +), MUM1 (plasma cell +), Bcl-2 (non-germinal center +), SOX-11 (-), CD30 (-), CD3 (T cell +), CD5 (T cell +), CD21 (FDC network +), CD23 (FDC network +), Ki-67 (+).

Systemic involvement and diagnostic reassessment: Despite biochemical evidence of activation of the hypothalamic–pituitary–gonadal axis, the patient presented with low body weight, delayed bone age, and reduced growth velocity, indicating that her growth retardation could not be explained by an isolated endocrine disorder alone. Instead, nutritional compromise and an underlying systemic disease process were considered likely contributors. Baseline laboratory investigations revealed hypoalbuminemia, dyslipidemia, and persistent proteinuria (Table 1), suggesting systemic metabolic disturbance and renal involvement. In addition, physical examination and imaging studies demonstrated generalized lymphadenopathy involving the cervical, axillary, supraclavicular, and inguinal regions. Abdominal computed tomography showed splenomegaly, and chest computed tomography revealed mild subpleural inflammatory changes. Taken together, these findings were more consistent with a systemic inflammatory or autoimmune disorder than with a primary endocrine cause. Given the multisystem abnormalities, an expanded autoimmune evaluation was performed (Table 2). Immunological testing demonstrated positive antinuclear antibodies, elevated anti-double-stranded DNA antibodies, decreased complement levels, lymphopenia, and increased 24-hour urinary protein excretion, supporting systemic autoimmune activation. Cervical lymph node biopsy showed lymphoid follicular hyperplasia with prominent plasma cell infiltration, without evidence of malignant lymphoproliferative disease. Pathological diagnosis of cervical lymph nodes is shown in Figure 2. These histopathological findings supported autoimmune-associated lymphadenopathy and helped exclude lymphoma. Based on the above clinical, laboratory, and pathological findings, the patient fulfilled the 2019 European League Against Rheumatism/American College of Rheumatology classification criteria for systemic lupus erythematosus (15) and was diagnosed with childhood-onset systemic lupus erythematosus. During hospitalization, she developed fever and rash, and her Systemic Lupus Erythematosus Disease Activity Index 2,000 score (16) was 7, indicating moderate disease activity. Renal biopsy was not performed; therefore, the histological class of lupus nephritis could not be determined. However, persistent proteinuria together with hypoalbuminemia suggested clinically relevant renal involvement and warranted close longitudinal monitoring.

**Table 2:** Autoimmune laboratory findings at hospitalization and 6-month follow-up.

Investigation	At hospitalization	6 months	Reference range
ANA (IgG)	Positive	Positive	–
ANA pattern 1	Cytoplasmic granular	Cytoplasmic granular	–
ANA titer 1	1:1000	1:1000	–
ANA pattern 2	Nuclear granular	Nuclear granular	–
ANA titer 2	1:320	1:320–1:1000	–
Anti–dsDNA antibody	280 IU/mL	–	<100
Anti-Sm antibody	Positive	–	–
Anti-RNP/Sm antibody	Positive	Weakly positive	–
Anti-SSA antibody	Positive	Weakly positive	–
Anti-SSB antibody	Negative	Negative	–
Anti-ribosomal P antibody	Positive	Positive	–
Anti-mitochondrial M2 antibody	Weakly positive	–	–
ESR (mm/h)	79	16	0–20
Rheumatoid factor	<11.3 IU/mL	–	0–20
Anti-CCP antibody	1.2 U/mL	–	0–5
CRP (mg/L)	10.16	<3.41	0–6
IgG (g/L)	16.9	10.9	8.6–17.4
IgA (g/L)	2.4	2.13	1–4.2
IgM (g/L)	2.83	0.97	0.5–2.8
IgE (IU/mL)	174	114	0–100
Complement C3 (g/L)	0.423	0.758	0.7–1.4
Complement C4 (g/L)	<0.07	0.11	0.1–0.4
Anti-cardiolipin IgM	25.43	–	0–20
Direct Coombs test	Negative	–	–
Anti-RA33 antibody	>200	–	<25
24-hour urine total protein (mg/L)	156	63	–
24-hour urinary protein excretion (mg/24 h)	187	45	–
Albumin (g/L)	30.5	44.8	42–56
Total protein (g/L)	61.2	72	68–88

ANA, antinuclear antibody; dsDNA, double-stranded DNA; Sm, Smith antigen; RNP, ribonucleoprotein; SSA, Sjögren syndrome A antigen; SSB, Sjögren syndrome B antigen; ESR, erythrocyte sedimentation rate; CRP, C-reactive protein; Ig, immunoglobulin; CCP, cyclic citrullinated peptide.

Following the diagnosis of childhood-onset systemic lupus erythematosus, immunosuppressive therapy was initiated promptly to control disease activity. Because the patient presented with systemic manifestations, including fever, rash, and marked lymphadenopathy, and had a SLEDAI-2 K score of 7, intravenous methylprednisolone (40 mg/day for 3 consecutive days) was administered as initial therapy to achieve rapid control of acute inflammation. After treatment initiation, the fever and rash resolved, and the lymphadenopathy decreased. Autoimmune laboratory findings at hospitalization and at 6-month follow-up are summarized in Table 2.

After discharge, treatment was transitioned to oral prednisone at 1 mg/kg/day for maintenance therapy. Given the potential adverse effects of prolonged glucocorticoid exposure on growth and skeletal maturation, the prednisone dose was adjusted during follow-up to limit treatment-related effects on growth and development while maintaining disease control. Hydroxychloroquine (5 mg/kg/day) was added as a steroid-sparing agent. During follow-up, the patient showed no evidence of ongoing disease activity, including fever, rash, mucosal ulcers, or joint symptoms. However, significant hair loss developed during the third month of treatment. Hydroxychloroquine was therefore discontinued, and mycophenolate mofetil (500 mg twice daily) was introduced as adjunctive immunosuppressive therapy. Subsequent follow-up showed progressive clinical and laboratory improvement. Inflammatory activity was effectively controlled, as reflected by decreases in CRP and ESR, while complete blood count values remained within the reference range. Immunological evaluation at 6 months showed persistent ANA positivity, relatively stable anti-dsDNA levels, and improvement in complement C3 and C4 levels, indicating partial control of immune activity. However, mild proteinuria persisted, with 24-hour urinary protein excretion remaining above the normal range, suggesting ongoing mild renal involvement and the need for continued monitoring. Nutritional status also improved, as evidenced by a marked increase in serum albumin, while immunoglobulin levels remained within the reference range despite minor fluctuations. The trends in height, weight, and BMI before and after treatment are shown in Figure 1.

## Discussion

This case illustrates an uncommon but clinically important presentation of childhood-onset systemic lupus erythematosus (cSLE), in which growth retardation and delayed puberty were the initial manifestations. In contrast to the typical presentation of cSLE, which more often includes malar rash, arthritis, fever, or overt lupus nephritis (17, 18), this patient first presented to an endocrinology clinic because of progressive growth deceleration and absent pubertal development. The diagnostic turning point was the discordance between minimal external pubertal signs and biochemical evidence of pubertal axis activation. Although Tanner staging showed B1P1 and bone age was delayed, the triptorelin stimulation test demonstrated a pubertal LH response, and pelvic ultrasonography showed uterine and ovarian development consistent with estrogen exposure. Once these endocrine findings were interpreted together with low BMI, hypoalbuminemia, persistent proteinuria, generalized lymphadenopathy, splenomegaly, positive ANA, elevated anti-dsDNA antibodies, and hypocomplementemia, the diagnostic framework appropriately shifted from a primary endocrine disorder to a systemic autoimmune disease. This case therefore illustrates that, in adolescents with unexplained growth retardation or delayed puberty, especially when endocrine findings do not fully explain the phenotype, clinicians should actively search for extra-endocrine clues rather than limit evaluation to the endocrine axis alone.

Physical development in children should be assessed as the integrated progression of somatic growth and pubertal maturation. In the present case, the principal problem was growth retardation with delayed puberty. Her birth history and early developmental milestones were unremarkable, and her earlier growth pattern did not suggest longstanding constitutional thinness or a lifelong growth disorder. Instead, serial anthropometric data showed that height velocity gradually declined after 6–7 years of age, while weight gain plateaued and BMI fell by the time of presentation. These findings emphasize that evaluation of pediatric growth should not rely solely on a single cross-sectional height or weight measurement, but should incorporate longitudinal growth trajectories, BMI, bone age, pubertal staging, and parental target height. In this patient, such an integrated assessment was essential for recognizing that the growth abnormality was acquired and clinically significant (19).

A major issue in this case is the interpretation of malnutrition. Undernutrition is a well-recognized cause of impaired linear growth and delayed pubertal progression, but in this patient it was more likely secondary than primary. Before disease manifestation, her height and weight were within an acceptable range during early childhood, whereas at presentation she had low BMI, hypoalbuminemia, dyslipidemia, and proteinuria. This pattern does not support longstanding primary nutritional deprivation. Rather, it suggests acquired nutritional compromise in the setting of active systemic disease, likely mediated by chronic inflammation, reduced intake, increased metabolic demand, and renal protein loss. This interpretation is better supported by the subsequent course: after treatment, albumin increased, inflammatory markers improved, and weight gain gradually recovered. In pediatric SLE, malnutrition may arise from chronic inflammation, reduced appetite, increased metabolic demand, and protein loss, particularly when renal involvement is present (20). Thus, in this patient, low body weight should be interpreted as part of the disease-related growth phenotype rather than as an isolated nutritional diagnosis; otherwise, the patient may be mislabeled as having simple nutritional delay and the underlying autoimmune disorder may remain undiagnosed. The literature also supports that poor growth in cSLE is multifactorial and may reflect the combined effects of chronic inflammation, suboptimal nutrition, delayed puberty, and treatment exposure (21).

Although cSLE should be considered only a rare cause of growth retardation and delayed puberty, the literature supports that growth impairment is a meaningful and underrecognized complication of pediatric lupus (7). Previous studies have shown that growth in cSLE may be adversely affected by chronic inflammation, pubertal timing, prolonged disease duration, and glucocorticoid exposure (8, 9, 22). Available data also suggest that approximately 15%–25% of affected children may fail to achieve their genetically predicted adult height, particularly when disease onset occurs around the pubertal period (10), and delayed pubertal maturation has also been reported, particularly in girls (23). In the Thai cohort reported by Ponin et al., approximately one-fourth of patients with cSLE had growth impairment, and longer disease duration before the late phase of puberty together with higher cumulative corticosteroid exposure were important predictors of poor growth outcome (21). Similarly, the Oman longitudinal cohort reported growth failure in 32% of children and identified pre-existing growth failure, higher cumulative steroid dose, and longer disease duration as important contributing factors (24). These observations are highly relevant to the present patient, whose disease became clinically evident during the years immediately preceding puberty. The mechanisms are multifactorial. Chronic systemic inflammation may disrupt the growth hormone–insulin-like growth factor 1 axis (8, 9, 25) and impair normal skeletal maturation, while hypothalamic–pituitary–gonadal axis dysfunction may delay pubertal progression (8, 9). In addition, chronic disease-associated anorexia, gastrointestinal dysfunction, and undernutrition may further compromise linear growth (8, 9, 20). Therefore, although cSLE should only be considered a rare etiology in the differential diagnosis of short stature or delayed puberty, it deserves attention when these features are accompanied by proteinuria, lymphadenopathy, immune abnormalities, or other extra-endocrine findings that are not explained by a primary endocrine disorder.

The follow-up course further supports a relationship between disease control and partial recovery of growth. At diagnosis, the patient had moderate disease activity, with a SLEDAI-2 K score of 7. After immunosuppressive treatment, her systemic condition improved, lymphadenopathy regressed. Serum albumin levels, urinary protein excretion, inflammatory markers, and complement levels also improved. Over time, weight gain and annual height velocity also recovered, and serial height, weight, and BMI trajectories showed gradual improvement after treatment. These findings suggest that at least part of the growth impairment was reversible and related to active inflammation and acquired undernutrition rather than to a fixed primary endocrine defect. However, improvement in disease activity does not guarantee immediate normalization of growth and puberty. In children with cSLE, catch-up growth may remain incomplete because of prior inflammatory burden, delayed skeletal maturation, and treatment-related effects, particularly glucocorticoid exposure (21–23). For this reason, management should aim not only for remission or low disease activity, as recommended by EULAR (26), but also for preservation of growth potential through steroid-sparing strategies, nutritional support, and longitudinal monitoring of height, weight, BMI, Tanner stage, and bone maturation.

## Conclusion

In summary, this case demonstrates that growth retardation and delayed puberty may be an early and misleading presentation of childhood-onset systemic lupus erythematosus. Although cSLE is a rare cause of growth impairment and pubertal delay, it should be considered in adolescents with unexplained short stature or delayed puberty, particularly when accompanied by proteinuria or immunological abnormalities. Early diagnosis and multidisciplinary management are essential to improve long-term growth and overall clinical outcomes.

## Data Availability

The original contributions presented in the study are included in the article/Supplementary Material, further inquiries can be directed to the corresponding authors.
